# Combining Residual Insecticide Spraying Campaigns with Targeted Detection and Specific Chemotherapy for *Trypanosoma cruzi* Infection in Children

**DOI:** 10.1371/journal.pntd.0000168

**Published:** 2007-12-26

**Authors:** Ricardo E. Gürtler

**Affiliations:** Department of Ecology, Genetics and Evolution, Universidad de Buenos Aires, Buenos Aires, Argentina; Fundação Oswaldo Cruz, Brazil

## Background

With 10–15 million of people infected with *Trypanosoma cruzi* (Kinetoplastida: Trypanosomatidae) and many more exposed to risk of infection, the burden of Chagas disease in Latin America amounts to as much as 2.7 times the combined burden of malaria, schistosomiasis, leishmaniasis, and leprosy in 2002 [1]. Following a short, mostly subclinical acute phase and a very long asymptomatic phase with very low parasitemia, 25%–40% of infected humans develop chronic disease with cardiac, digestive, or neurologic manifestations that leads to a reduced life span [2]. Human transmission of *T. cruzi* is mediated by nearly a dozen blood-sucking species of triatomine bugs that infest resource-limited, rural houses and their outbuildings, but it may also occur by blood transfusions and from infected mothers to their children.

A series of intergovernmental control initiatives led by the Pan American Health Organization were launched in Latin America during the 1990s with the objectives of eliminating transmission of *T. cruzi* by blood transfusion and by domestic populations of triatomine bugs by the year 2010 [2],[3]. Control actions reduced the geographic range and infestation prevalence of major triatomine vectors and led to the interruption of transmission in Uruguay, Chile, and Brazil, and to significant improvements in Central America and elsewhere [2],[3]. However, active vector-borne transmission persists in vast areas of Argentina [3],[4], El Salvador, and Colombia, among others; and several countries (Mexico, Peru, Colombia, and Costa Rica) have no national programs for the control of Chagas disease vectors [5]. The growing decentralization of vector control operations to the provincial and municipal levels since the early 1980s added the still-unmet challenge of coordinating efforts among districts differing in infestation, control status, resources, and priorities, and between national, provincial, and municipal public health levels [6].

Large-scale screening of blood donors in Latin America began in the 1980s following the emergence of AIDS, and currently is in place in nearly all countries, though at differing coverage rates. These programs brought about a significant reduction in the prevalence of *T. cruzi* infection among blood donors in most of the region [7]. Although most advances in the safety of the blood supply since 1993 originated from increased screening coverage for infectious diseases and better quality assurance, it has been estimated that tainted blood may have caused *T. cruzi* infections in 12 of 17 Latin American countries over the period 2001 to 2002 [7]. With the sustained wave of immigration from Latin America to US, Canada, and Western Europe, transplant- and transfusion-related cases of Chagas disease jointly with congenital cases have been reported more frequently in the target destinations.

Chagas disease control programs traditionally have focused on interrupting vector- and blood-borne transmission of *T. cruzi* rather than on active case detection and specific treatment of infected people. One reason for this is that the only available drugs for specific treatment of *T. cruzi* infection, nifurtimox (since 1967) and benznidazole (since 1972), were traditionally considered effective only during the acute phase of infection or shortly after it. This notion persisted until two randomized clinical trials, conducted with support from the World Health Organization's Special Programme for Research and Training in Tropical Diseases (TDR) and national agencies during the 1990s, demonstrated that most infections treated with benznidazole during the early chronic phase (i.e., in seropositive children aged ≤12 y with unknown duration of infection) could be cured [8]–[11]. In the meantime, experimental studies changed the view that Chagas disease is primarily an autoimmune response toward one that the disease is a problem of parasite persistence [12]. In addition, the progress in vector control status achieved during the last decades has paved the way to conceiving the specific treatment of *T. cruzi*-infected children residing in traditionally endemic rural settings (e.g., Argentina, Bolivia). More recently, specific treatment has been extended to seropositive children aged ≤15 y in some countries, and is being offered more frequently to adults with long-term chronic infections because it might moderate disease progression [13]. Because individuals in the chronic stage display a long-lasting serological response to *T. cruzi* infection after specific treatment, in some cases demonstration of cure by means of conventional serological methods may take more than 10 y [9]. The efficacy of anti-trypanosomal drugs apparently decreases with the duration of infection, whereas their adverse effects increase with age and occasionally may be serious if treatment is not discontinued and proper care given [14],[15]. As the new study by Levy et al. [16] published in *PLoS Neglected Tropical Diseases* stresses, “…without timely diagnosis, children infected [with *T. cruzi*] prior to implementation of vector control often miss the window of opportunity for effective chemotherapy.”

Another important reason for the observed low rates of specific treatment of children seropositive for *T. cruzi* is that health services and Chagas disease control programs in Latin America lack or do not allocate sufficient resources for comprehensive serological screening and supervised treatment in the most affected endemic areas. There, health services usually are understaffed and overburdened by competing demands, not the least of which is to combat domestic reinfestation after massive insecticide spraying campaigns [4]. The usual approaches to active case detection require surveying the whole population at risk (in house-to-house or school-based surveys), and therefore are labor-intensive and costly. It is in this context that the article by Levy et al. [16] attains high relevance for the pending task of massive case detection and treatment of infected children in resource-poor settings.

## A new study on targeted control strategies for Chagas disease

Michael Levy et al. [16],[17] describe for the first time the emergence of *T. cruzi* transmission in an urban or periurban environment, and its possible epidemic spread from one or several points of parasite introduction in a geographically defined area in the city of Arequipa, Peru. The primary aim of the study was to develop targeted screening strategies to detect *T. cruzi* infection in children from data collected during a vector control campaign directed against the major vector *Triatoma infestans*. Although household clustering of *T. cruzi* infection and vector infestation has long been known [18]–[20], the researchers are also the first to describe the spatial aggregation of seropositive children within looser clusters of infected vectors.

The researchers took a simple and direct approach to detecting infected children. They accompanied vector control program operations in one community to collect entomological, demographic, and environmental data as residual insecticide spraying was carried out at each geo-referenced household; they then performed a cross-sectional serological survey for *T. cruzi* infection among children aged ≤18 y and mapped out the occurrence and densities of vectors, infected vectors, and seropositive children. By using spatial analysis and multivariate Bayesian modelling techniques, the researchers identified clusters with children at high risk of infection for targeted screening and treatment, and evaluated the benefits of alternative screening strategies.

Among the main study findings, child infections were geographically clustered and apparently occurred at all vector density levels. Significant spatial clustering of seropositivity among children occurred up to 270 m of an identified case. A key result from the modeling effort is that 83% of infected children could be identified while testing only 22% of eligible children. The researchers then devised a two-step screening strategy that, for the first step, begins by ranking children based on their age and the relative density of vectors captured within their houses (preliminary screening), and then examines for infection a proportion of the children predicted to be at highest risk. In the second step of screening, the information on the detected seropositive children is used to identify and test other children living within given distances from the former (ring screening). As in earlier studies [18],[19],[21], child seropositivity was significantly associated with domestic vector densities and child age; additional information on whether domestic *T. infestans* were infected with *T. cruzi* (involving laborious procedures) did not improve the ability of the model to predict child infection. The article also reminds us of the outstanding ability of the vector to infest urban environments with substandard housing and transmit *T. cruzi* to humans.

## Strengths and limitations of the study

Major strengths of the Levy and colleagues study may be found at levels that encompass study design and careful data collection in a well-defined area, to data analysis with sophisticated statistical methods and cautious interpretation of findings.

Lack of demographic and behavioral data limit the interpretation of results to some extent. Additional information on the individual timing of settlement (including birthplace, residence period, and travel history) and the main geographic sources of immigration would contribute to a better understanding of the transmission system and could help corroborate whether all child infections were vector-borne and autochthonous, as the spatial analysis and the identified vector-related predictors suggest. Although most of the study children were born in Arequipa, those in the older age group (close to 18 y old) and elder family members likely became infected elsewhere, given that they were rural immigrants relocated to periurban settlements from 1980 to 1995 to escape from terrorism [17]. This detail does not conspire against the primary aim of the study (i.e., identifying the infected children regardless of the origin of the infection), but it affects more refined elaborations of the relationship between risk of infection, vector densities, and transmission thresholds. Measurement of transmission thresholds is fraught with several sources of inaccuracy [21], and in the best case they might only be achieved through a prospective study.

As the authors acknowledge, individual information on migration, the participants' maternal seropositivity status, history of previous blood transfusions, and householders' vector control practices would be most valuable, and may explain some of the cases missed by the models. For research purposes and refinement of the models, collecting data on potential predictors that may increase the models' ability to identify the locations of infected children may help reduce the fraction of infections lost to detection and subsequent treatment. This is a crucial point related to equity, because in the absence of subsequent screening instances the infected children lost to detection would also lose the (currently suggested) window of opportunity for effective specific treatment of *T. cruzi* infections. Such a window of opportunity is itself a matter of controversy [11],[13],[15],[22]. The utilization of highly sensitive rapid (dipstick) tests for detecting antibodies to *T. cruzi* in finger-prick blood samples may simplify and speed up the screening stage at a moderate net cost relative to standard laboratory-based diagnosis of serum samples drawn by venipuncture [23]. Increasing the sensitivity of the screening models at the expense of its specificity is clearly indicated as the next step. Replication of the targeted approach to detection and treatment in communities that have recently experienced higher levels of transmission, and therefore have a larger number of infected children to identify and treat, would be very useful.

## Implications of the study for Chagas disease control

Levy et al. [16] raise two subjects that are rarely debated in the field of Chagas disease control: the optimal use of limited resources, and the integration of case detection and treatment of children into disease control programs that traditionally have focused on vector control. Mathematical modelling also supports the hypothesis that vector control combined with specific treatment is highly cost-effective compared with vector control alone [24]. Lack of integration between both components entails lost opportunities for improved disease control.

A major contribution of this article is the identification of “hot spots” of infestation and transmission within an apparently homogeneous community. The identification of such “hot spots” would not only enable more targeted case detection and prompt treatment, as the authors emphasize, but it may also contribute to improved prevention of transmission after residual insecticide spraying [25]. *T. cruzi* infection in domestic dogs and cats was highly aggregated at the household level and fell close to the 80/20 rule [26], which states that a small fraction (≤20%) of the households makes a disproportionate contribution (≥80%) to infection prevalence [27]. Moreover, the infectiousness of domestic dogs seropositive for *T. cruzi* to triatomine bugs was also highly aggregated at the population level [28]. Targeted case detection and treatment combined with selective vector control would not only increase the impact and cost effectiveness of the control program, but it could also help increase its public acceptance and long-term sustainability, as observed in a long-term prospective study in northern Argentina [4].

It is beyond dispute that benznidazole and nifurtimox should be more widely used for specific treatment of *T. cruzi* infection in children at all stages of the disease. Although the supply of these drugs has recently improved [5], access to them in some endemic settings remains problematic. In traditionally endemic settings where reinfestation is recurrent, vector surveillance and control systems need to be established or strengthened before specific treatments are made available more widely. Beyond targeted detection, sustainable vector surveillance, and better access to drugs, we still need to increase awareness of treatment opportunities in the medical sector serving endemic settings and in the affected population groups, and to train local physicians in the supervised treatment of children seropositive for *T. cruzi*.
